# Mental Healthcare Practices from Entry to Release across Southeastern Jails

**DOI:** 10.21203/rs.3.rs-4144413/v1

**Published:** 2024-03-28

**Authors:** Elena DiRosa, Tonya Van Deinse, Gary Cuddeback, Andrea Murray-Lichtman, Jessica Carda-Auten, David Rosen

**Affiliations:** University of North Carolina at Chapel Hill; University of North Carolina at Chapel Hill; Virginia Commonwealth University; University of North Carolina at Chapel Hill; University of North Carolina at Chapel Hill; University of North Carolina at Chapel Hill

**Keywords:** Mental health, jail research, incarceration, healthcare practices

## Abstract

**Background:**

Individuals with mental illnesses are disproportionately incarcerated in jails, which have become *de facto* mental health institutions across the US. Yet there is limited research describing mental healthcare practices from entry to release among multiple jails and states.

**Methods:**

We conducted 34 semi-structured interviews with jail healthcare personnel across five Southeastern states.

**Results:**

We report results on challenges and practices related to mental health staffing, screening, additional evaluations and services, and discharge planning in jails. Initial mental health screenings were often restricted to the detection of suicidality and history of treatment and medications as opposed to current mental health symptoms. Use of validated mental health screening forms was uncommon. We found delays in care between the initial health screening and being evaluated by a mental health professional. Most jails reported primary responsibilities for mental health care as preventing suicides and managing psychiatric medications. Jails reported mental health care as challenging to manage, with high volumes of individuals with mental health needs, yet limited resources, especially regarding staffing. Discharge planning was limited despite reports of poor continuity of mental healthcare.

**Conclusions:**

Jails have a constitutional duty and opportunity to provide adequate healthcare to individuals with mental illnesses, yet practices are insufficient and resources are limited across jails. Based on our findings, we recommend 1) greater adoption and revisions of jail health standards 2) system improvement that expands identification of mental illnesses and quicker, less variable follow-up mental health evaluations, 3) improved linkages and supports for community resources that prevent incarceration of this population.

## Background

Individuals with mental illnesses are disproportionately incarcerated in US jails, which have become de facto mental health institutions. Higher rates of incarceration in jails have been linked to insufficient community mental health resources (1), and findings point to nearly ten times as many individuals with serious mental illnesses incarcerated in jails and prisons than in state psychiatric hospitals (2, 3). In a national survey of jailed persons, 44% had a mental health condition including major depression, bipolar disorder, schizophrenia, and post-traumatic stress disorder (PTSD), and 26% met the threshold for “serious psychological distress,” compared to just 21% and 5.6% of the general population, respectively (4, 5). From 2006 to 2019, suicide was the leading cause of death for individuals incarcerated in jails, indicating that too often acute mental health needs remain unmet in jails (6). Moreover, incarcerated individuals with mental illnesses are more likely to be re-arrested after being released into the community, perpetuating pernicious cycles of medical and social instability (7–9).

Given the high prevalence of mental illnesses in jails and jails’ legal responsibility to provide healthcare (10), a comprehensive understanding of mental health services in jails is needed. However, much of the research literature surrounding this issue is focused on prevalence estimates of mental illnesses among those in jail (11, 12) or describe healthcare services within a single jail or within jails from a single state (13–15). Alternatively, existing studies surveying personnel from multiple jails often include only the perspectives of jail administrative staff at the exclusion of healthcare personnel (16, 17).

The limited research describing jail mental health services suggests the following: across jails there are vast differences in available care (16–18) and mostly limited onsite mental health care, particularly in smaller jails (18); mental health screening occurs in most jails but the use of validated mental health screening forms is rare (16, 19); there are delays in care between initial screening and mental health evaluation (17); and there are deficient protocols for discharge planning for individuals with mental illnesses (15, 20). Studies examining jail mental health practices have not addressed the full continuum of services from entry to release and studies focusing on jails in the US South, which have the highest rates of incarceration in the United States (17, 21), are uncommon.

Given these gaps in the literature, this article reports on results from a qualitative study examining challenges and practices related to mental health staffing, screening, additional mental health evaluations and services, and discharge planning for incarcerated individuals with mental illnesses across multiple jails and states.

## Methods

### Study Design

This article reports on a subset of mental healthcare-related findings from qualitative interviews conducted with jail personnel who participated in a larger study of correctional healthcare within jails across five states in the Southeastern United States [citation blinded for submission]. All research activities were approved by the University of [blinded for submission] Institutional Review Board.

### Study Population and Data Collection

The research team compiled a list of county and regional jails in 5 states—Alabama, Georgia, North Carolina, South Carolina, and West Virginia—based on the 2006 and 2013 Bureau of Justice Statistics (BJS) Census of Jails (22, 23). This list was updated with information about jail openings and closures obtained from Internet searches. In total, the research team identified 346 jails, 96.8% of which were county jails and 3.2% were regional jails. The research team stratified the 346 jails by urbanicity (i.e., urban, rural, completely rural; (24), jail size as reported by BJS COJ (i.e., small, medium, large), and geographic location within state.

The goal of study enrollment was to recruit participants from a diverse sample of jails across the strata described above. Recruitment from potential study jails was initially informed by suggestions from local and regional stakeholders, and in NC, the study team initially contacted personnel at jails who had been responsive to another study. In many instances, jails were contacted by “cold call.” In all cases, the team conducted phone-based outreach to prospective jails to identify the person “most knowledgeable” about day-to-day medical operations. Once potential participants were identified, staff explained the purpose of the study and provided a study fact sheet by email. Potential participants able and willing to provide their informed consent were enrolled in the study.

Study recruitment, interviews, and analyses were conducted iteratively, continuing until saturation of key themes was achieved. Through this process, the team eventually contacted 125 jails. Among the 125 jails, personnel from 34 jails agreed to participate and 14 declined. For the 77 out of 125 jails that did not participate, personnel were unresponsive to the team’s outreach or, for a variety of reasons (e.g., scheduling, no-shows), the interview could not be conducted. Across the 34 jails, 38 individuals took part in a semi-structured interview with four jails electing to have two staff members interviewed. The final sample of interviews consisted of between 6% and 18% of jails from each state.

Interviews were conducted between August 2018 and February 2019 and typically lasted 60 to 90 minutes. Of the 34 interviews, 27 were conducted onsite in a private room at the jail, and the remaining were conducted by phone. Two researchers with extensive training in qualitative methods and correctional health conducted interviews.

### Interview Guide

The semi-structured interview guide for the “parent” study [citation blinded for submission] consisted of 60 items that examined healthcare resources, provision of services, and screening and assessment processes. The current study focused on interview responses addressing mental health screening, additional mental health evaluations and services, and discharge planning, as well as participants’ answers to the question: “What are the struggles you commonly encounter in managing mental health conditions?” See Appendix A for the complete interview guide.

### Data Analysis

The research team used the Framework Method to guide the analysis (25). Team members independently read and created memos for each interview. The research team then developed an initial codebook organized by interview questions and *a priori* themes. The research team tested the codebook on a sample of interview transcripts, adjudicated any coding inconsistencies, and edited the codebook until final agreement was reached. Each transcript was then independently coded by two team members and discrepancies were discussed until reaching consensus. After coding, the research team summarized results for each code, creating a spreadsheet-based matrix of study themes and subthemes. Relevant excerpts from code reports were entered in matrices such that each row represented one jail. The research team used this matrix of themes to describe and compare findings across jails. Additionally, some analyses were conducted by jail size, although trends in the data were identified rather than conducting the analysis by the three categories used to sample.

## Results

Results are organized into five themes: (1) limited onsite mental health staffing and resources; (2) mental health screening processes; (3) additional mental health evaluations and services; (4) challenges caring for individuals with mental illnesses; and (5) discharge planning services. In this section, we describe findings common among participating jails and include notable exceptions and differences by jail size. Quotes representative of key findings are followed by a study identification number and jail size. Additionally, though the article reports findings by jail, participants were individual staff members and results should be interpreted as reflecting participants’ views of jail operations rather than jail operations themselves. In all cases, including those where participants report findings, the unit of analysis is the jail, not the participant.

### Study Sample

Most study participants described their role as either nurse or healthcare administrator (*n* = 30) and the remaining participants were made up of regional health care managers (*n* = 4) and jail administrators (*n* = 4). Of the 35 participants queried about their work history, most (*n* = 30) had been in their positions for at least one year (Table 1).

Of the 34 jails, 18% (*n* = 6) were large, 62% (*n* = 21) were medium-sized, and 21% (*n* = 7) were small. Half (*n* = 17) of the study jails were in urban counties, and half were either in rural (*n* = 15) or completely rural (*n* = 3) counties. In our sample, large jails were typically located in urban counties and small jails were located in rural counties (Table 2).

Most medium-sized jails with fewer than 200 incarcerated individuals were located in rural counties and most medium-sized jails with greater than 200 incarcerated individuals were located in urban counties. About one quarter of jails (*n* = 9) were accredited by the National Commission on Correctional Healthcare (NCCHC) or American Correctional Association (ACA), and nearly 20% (*n* = 6) of jails were described as being over-capacity at the time of the interview.

### Limited Onsite Mental Health Staffing and Resources

Jails reported limited onsite mental health staffing and resources. Below are key findings characterizing these limitations.

### Limited Onsite Mental Health Staffing

Most jails reported considerable limitations in onsite mental health staffing resources and availability. A description of onsite mental health staffing and supplementary mental health services (e.g. telemedicine, community, and prison system resources) is provided below and summarized in Table 3.

Among all study jails (*n* = 34), participants in nearly half (*n* = 15) reported the absence of routine onsite mental health staffing of any kind (e.g., counselors, psychiatric prescribing providers; Table 3). [Insert Table 3.] Of the remaining jails (*n* = 19), most (*n* = 13) had onsite staffing at least 2 times per week, and six jails had onsite staffing once per week.

Most jails specifically reported that medication management staffing was limited. In fact, most jails (*n* = 25) reported that any onsite prescribing mental health provider (e.g. psychiatric nurse practitioners or psychiatrists) coverage occurred once a week or less, or was nonexistent. A quarter of jails (*n* = 9) reported managing such limitations by having a psychologist or mental health counselor evaluate individuals and make medication recommendations to a prescribing provider not specifically trained in psychiatric medication prescription (e.g., physician, physician assistant, or nurse practitioner). One participant noted this type of scenario:

Every Thursday he does regular patient, like CB—cognitive behavior therapy…He can’t prescribe. He’s a social worker, so he can suggest, maybe you might wanna look at antidepressant. Then our doctor looks at him, reviews him, and says, “Okay, maybe he does need to be on antidepressant.” (17, Medium-sized jail)

Conversely, the minority of jails (*n* = 10) reported regularly scheduled onsite prescribing mental health provider availability for psychiatric medication management and referrals (Table 3). Most jails with prescribing mental health provider availability (*n* = 9) reported availability at greater than once a week.

Onsite counseling staffing was also reportedly sparse. Specifically, most jails (*n* = 21) reported that onsite counseling staff coverage was limited to once a week or less or was nonexistent (Table 3). One participant explained a typical counseling schedule: “[our mental health counselor] comes once a week…it’s not even a day. She’s only here three or four hours a week.” (34, medium-sized jail). Almost half of jails (*n* = 15) reported no onsite mental health counseling coverage or coverage occurring only on an as-needed basis (e.g., for court evaluations) or in crisis circumstances (e.g., suicide assessments). The majority of jails (*n* = 18) with limited or no onsite mental health counseling tended to be jails with capacities of 200 or fewer incarcerated individuals.

Though less common, some jails had more extensive mental health counseling coverage. While participants from most jails (*n* = 18) reported some onsite mental health counseling staff (e.g., psychologist, social worker, or therapist) availability, only about a third of overall jails (*n* = 11) reported onsite counseling coverage at greater than once a week (Table 3). The majority of such jails (*n* = 9) with full-time or nearly full-time onsite mental health counseling tended to be larger jails with a capacity of more than 500 incarcerated individuals. One participant discussed the benefit of weekly group counseling in one jail:

We have outside people [who] come in and offer classes…They do one-on-one counseling with them [and] groups maybe twice a week…It really does good for them to get in those groups and be able to talk and get out of the cell and it kinda helps with morale…They’re always locked up and they look forward to the groups when they know it’s that time. (12, medium-sized jail)

Additionally, other jails reported supplementing onsite mental health care by relying on nurses and detention officers to manage high volumes of individuals with mental illnesses. For example, most jails (*n* = 33) reported that nurses (typically licensed practical nurses) were scheduled onsite more frequently than mental health staff and were typically the first healthcare staff to triage individuals identified as having a mental illness for additional evaluations and medication referrals (*n* = 18). Of all staff, detention officers were reported as having the most onsite coverage (*n* = 34), often monitoring individuals on suicide watch (*n* = 12), screening for mental illnesses (typically including suicidality; *n* = 25), triaging or passing sick calls to nurses (*n* = 9), and handling emergencies and urgent medical responses, including suicidality (*n* = 7). Even jails with daily mental health coverage reported depending on nurses for mental health care, as one participant described in more detail:

You kind of have to be a psych nurse to work in here…That’s the bulk of who you’re actually dealing with on a daily basis. Most of the nurses, all 20 nurses and all 18 LPNs, have got a real significant interest if not a specialty in psych. They don’t counsel or use it officially like that, but we are using it on a daily basis, every time we’re in front of a patient. (5, large-sized jail).

### Telemedicine

Some jails (*n* = 9) reported utilizing telemedicine for medication management or mental health counseling instead of, or in addition to, onsite staff, with most (*n* = 6) describing it as occurring once a week or less (Table 3). One participant described the value of telemedicine for counseling in one jail:

Then we have a doctor of psychology who does a mental health clinic once a week…The folks that have mental illness or anybody can request to talk to him. He manages that for us… Yeah…when they said, “We’re gonna have a doctor of psychology,” I thought, “Great.” They said, “We’re gonna do it by videoconference.” I kind of went, “Oh, this is not gonna work,” but it’s been wonderful. (4, medium-sized jail).

### Community and Prison System Resources

Other jails (*n* = 7) relied on offsite community mental health agencies to supplement a lack of onsite mental health care (Table 3). These jails reported either sending individuals out to agencies or calling an outside agency staff member to come onsite in case of an emergency. Coverage was often described as *ad hoc* and was used to address episodes considered to be psychiatric emergencies.

Furthermore, participants in most jails (*n* = 4) that relied on community agencies for mental healthcare reported challenges accessing the agencies services. The main challenges jails reported included difficulty scheduling, unwillingness to assist with care other than psychiatric crises, and deficient available staffing. One participant described a community agency that was largely unresponsive to requests for care:

Actually, we have a mental health facility that’s located in [town] about 30 minutes from here, and they come not too often. I’ve been here about four years, and I think I’ve seen them come probably less than ten times to see a patient…They’re not really cooperative with us…I’m just continuously calling, trying to get an appointment, and they just don’t return phone calls. (33, small-sized jail)

Other participants reported that individuals required better mental health services than their jails could provide. In one state, jails have access to a state program in which jails unable to provide sufficient health care onsite can, for a fee, transfer a limited number people to the state prison system for care. Half of study jails from this state (*n* = 6) reportedly used this program specifically for individuals with severe mental illnesses.

### Mental Health Screening Processes

**Figure 1.**
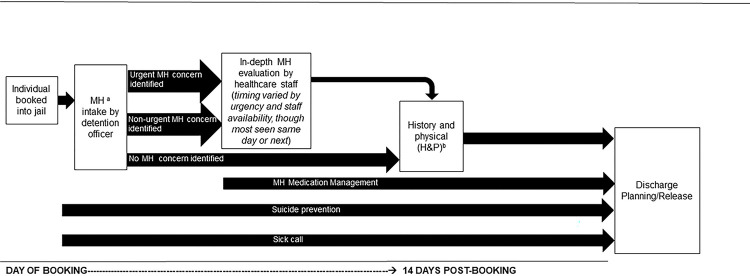
Most Commonly Described Processes for Mental Health Screening, Additional Evaluation, and Services. ^a^ MH = Mental Health ^b^ Assessment and medical care and H&P are conducted by medical staff (i.e. nurses or medical providers). *Note:* This figure depicts the most commonly described processes for mental health screening, additional mental health evaluations as determined by level of urgency, and services (medication management, suicide prevention, and sick call) available to individuals in jails until discharge planning/release. This figure would not represent processes for individuals released from jail before 14 days or earlier.

In order to identify individuals with mental health concerns, all jails (*n* = 34) reported screening individuals during the general intake screening process at booking. This screening was typically conducted within hours of an individual’s arrival. Key findings relevant to mental health screening are presented below and depicted in Fig. 1.

### Focus of Mental Health Screening

Most jails’ (*n* = 18) mental health screenings focused primarily on suicide risk, mental health treatment history, and current psychiatric medication prescriptions, but not on current psychiatric symptoms. As one participant described the lack of symptom assessment: “We really only ask three things. We ask if they’ve ever attempted suicide, if they see mental health outside, and if they’ve ever been hospitalized by mental health.” (20, medium-sized jail) In contrast, participants from about one quarter of jails (*n* = 9) reported assessing individuals’ current mental health symptoms or using a validated questionnaire to assess mental illnesses during the initial screening process.

### Administration of Screening Instrument

Most jails (*n* = 24) reported that detention officers were responsible for health screening at booking (Fig. 1).

One participant recounted the process:

[Detention officers] ask ‘em about their medical issues. Do you know if you have any mental health history? Do you have any drug abuse history?…It asks the officer for observations. Do they have any visible signs of injury? Are they acting bizarre? Any shortness of [breath]? Are they suicidal? Then they also have a whole suicide prevention screening that they do. (7, small-sized jail)

Although less common, other jails required healthcare staff to conduct this screening. About one quarter of jails (*n* = 9) reported that nurses or nursing assistants were responsible for the initial medical screening and that officers did not conduct any health-related screening. Most jail facilities that had healthcare staff conducting screenings had capacities of more than 600 individuals. One participant described a typical scenario:

The [officers] don’t do any kind of health screening…What they do is they book ‘em in and a sheet is produced. We pick up that sheet…and bring ‘em into our office, where we do the…mental health screening, and if something flags mental health…we’ll call Mental Health.” (22, large-sized jail)

In a couple of jails, initial mental health screening forms were sometimes self-administered by the individual getting booked into jail, either as part of a routine practice or when understaffed. One participant described how this could lead to incomplete forms: “It’s not really private, and sometimes I think they give the inmate this [screener] and let them [fill it out]. Sometimes you’ll be like, “What’s this mean?”…and that’s probably how half of them get missed.” (10, medium-sized jail)

### Additional Mental Health Evaluations and Services

#### Post-screening Follow-up Evaluations

Jails reported post-screening evaluation procedures for individuals identified as having a mental health concern and those not identified as having a mental health concern during the initial screening. Main findings are presented below and depicted in Fig. 1.

In almost all jails (*n* = 33), individuals identified as needing a follow-up mental health assessment after the initial screening were typically seen by onsite healthcare personnel for a more in-depth mental health evaluation (Fig. 1). This evaluation contained more extensive questions to further assess mental illness and provided an opportunity to acquire information that individuals might not have felt comfortable sharing during the initial screening.

Timing of this evaluation usually varied by staff availability and level of urgency (Fig. 1). Participants reported that urgent cases were often referred to a nurse, prescribing provider, jail supervisor (typically to authorize sending individuals to the emergency room), or mental health provider as soon as possible. Further, most jails (*n* = 24) reported that a prescribing provider or a counselor was available 24/7 via phone or telemedicine to discuss individuals deemed to be in crisis. The most commonly reported issue involved suicide risk. One participant described a typical protocol for assessing suicide risk:

[The detention officers] have a whole suicide prevention screening…If they score a certain number on [that], they’re gonna automatically notify their supervisor. The supervisor’s gonna notify the sergeant. The sergeant’s gonna notify the captain. The person’s gonna go into suicide watch immediately. Our provider who is on call will be notified. (7, small-sized jail)

Jails reported timing for less urgent mental health follow-up evaluations as more delayed and varying widely when compared to urgent concerns. Though timing was also dependent on staffing schedules and availability, most jails (*n* = 24) reported that individuals were typically seen by a nurse either on the same day or the next day or, with more delay, by a mental health provider (Fig. 1). One participant described a typical waiting process to see a mental health provider:

If they answer “yes” to certain questions, it’s gonna automatically generate an appointment to see mental health…but because mental health only comes once a week, we’ll reschedule it for that day that she comes…We try to let them see mental health as soon as possible, especially if they answer yes to one of those questions. (31, medium-sized jail)

There were some exceptions found to typical post-screening evaluation processes. Almost a quarter of jails (*n* = 8) reported that individuals needing a mental health evaluation received automatic referrals to an onsite mental health or medical provider right after the initial mental health screening. In some jails (*n* = 5), individuals could be seen for this evaluation up to 72 hours after booking. This usually occurred if the initial screening happened over the weekend and the nurse was not scheduled to work at the jail until Monday morning. Other jails (*n* = 9) reported either having no mental health staff available to evaluate individuals or that they were only available for emergencies.

In most jails when an officer did not identify a mental health concern during screening (*n* = 15), individuals could wait up to 14 days to see a healthcare staff member for a history and physical (H&P) exam, typically conducted by a nurse (Fig. 1).

Additionally, in all jails, individuals could request nursing or medical care at any point during their stay through a “sick call” request (Fig. 1). Half of jails (*n* = 17) reported that individuals requesting mental health care through a sick call were not typically charged a fee. In other jails, copays varied from $2 to $25 per visit. In about one quarter of jails (*n* = 9), officers triaged sick-call requests, judging their level of urgency. Further, officers were commonly described as the “eyes and ears” of housing areas. If an individual exhibited signs of psychiatric emergency or suicidality, officers were often responsible for alerting a supervisor or healthcare staff member, regardless of formal sick call submission.

### Services for Individuals Identified with Mental Health Conditions

Most participants reported that their jails’ primary responsibilities for mental health care were preventing suicides and managing psychiatric medications; relevant findings are presented below and depicted in Fig. 1.

Suicide prevention was reported as a central concern and liability for jails, with officers often playing an important role in identifying potential suicide risk. In most jails (*n* = 24), officers were responsible for detecting suicide risk at intake and some jails (*n* = 8) noted that officers were frequently on the lookout for risk of suicide in housing areas. In some jails (*n* = 14), detention officers were also responsible for observing and documenting progress for individuals being monitored for suicide risk. One participant described a typical process:

If we have an inmate saying that they’re at high risk of suicide…we would pretty much put him where he could be watched at all times, and I would have called mental health, and have them set up an appointment to have an assessment done on this inmate ASAP. Then we would have an officer watch the inmate. We’ll check on [him] every 10 to 15 minutes around the clock until we get the assessment done.” (25, small-sized jail)

Individuals found to be at risk of suicide were commonly referred to a healthcare or senior jail staff member to determine next steps. Until then, almost half of jails (*n* = 15) reported placing individuals at risk of suicide in a cell by themselves until they could be seen by a nurse, medical provider, or mental health professional. Other jails housed individuals in booking areas or cells with cameras (*n* = 5), medical units (*n* = 4), special housing for individuals with mental illnesses (*n* = 3), or with other individuals at risk of suicide, unless they were also homicidal (*n* = 2).

Most jails (*n* = 23) noted strict safety protocols for suicide prevention. Almost half of jails (*n* = 15) reported physical measures to prevent suicides, having individuals remove their clothes, removing access to plastic utensils, and using a suicide smock, often called a “pickle suit” or “turtle suit,” to restrict physical movement that could lead to self-harm. Some jails reported dressing individuals in paper clothing and giving them paper utensils and Styrofoam cups. One participant described this protocol in more detail:

When someone’s on suicide watch, they can have nothing with them except for the suicide smocks…They have the blanket, and all of it, you can’t rip…When they eat, they are given a paper spoon so that they can’t hurt themselves with that. They’re obviously watched, not only by the officers. They pull them up [on the cameras]. I watch them, and then we keep a running chart…every day, I go in, and we talk to them a couple of times, ask them how they’re doing. (30, medium-sized jail)

One participant noted that for extreme cases, individuals were placed in restraints and given one hour of free time out each day:

Some places have chairs. We basically fold [the person] in half in a sitting position, put a helmet on them, and the wrap restrains them like this so that they can’t hurt themselves… They get an hour of free time out. Usually that’s when they get a shower. (29, Medium-sized jail)

Other jails (*n* = 6) reported on staff uncertainty and doubts discerning suicidality, which participants sometimes recounted as a burden on jail staff. In fact, almost a third of jails (*n* = 11) reported placing all individuals with the potential for suicide risk on suicide watch despite not knowing if the individual was feigning suicidality or hiding the truth. One participant explained a common suicide watch tactic used to be cautious:

If something’s off, you don’t feel right, I tell the nurses, “Go with your gut. If they’re telling you yes to your face and answering all the questions they think you want to hear.” I said, “But you don’t feel comfortable, put them on suicide watch. You can always step them down, but you can’t bring them back.” (14, small-sized jail)

Other jails reported reservations related to individuals’ hopes of getting sent to the hospital for a “warm bed and television” or behaviors explained as merely “acting out.”

Although jail and healthcare staff were frequently allowed to place individuals on suicide watch, in the majority of jails (*n* = 19), only a doctor, nurse, or mental health provider could reportedly remove individuals from suicide watch. A little less than half of jails (*n* = 15) reported only allowing mental health staff or prescribing providers to remove individuals from suicide watch.

Medication management was also reported to make up a significant portion of mental health care. However, participants from most jails (*n* = 20) described either prohibiting or severely limiting controlled substances, and several jails (*n* = 8) reported substituting active mental health prescriptions with different medications. Reasons for these restrictions and substitutions included high cost or concerns about misuse. One participant noted such restrictions:

We don’t give any Trazadone. We don’t give any Seroquel. We don’t give BuSpar. We don’t give Xanax. We don’t give Valium for that. We use the Valium for the benzo detox and that’s it…We don’t do Wellbutrin. Wellbutrin has a very high street value. I cut all of those out. We don’t give Benadryl. We don’t give Vistaril.” (6, large-sized jail)

Multiple jails reported placing a higher priority on continuing rather than initiating mental health prescription medications. Almost half of jails (*n* = 16) reported providing mental health medications only for individuals maintaining an active prescription outside of jail, pending verification from an outside pharmacy or medical office. Even so, in almost a third of jails (*n* = 11), including cases when a medication could not be verified, individuals with active mental health prescriptions were required to be evaluated by a mental health or prescribing provider before medications could be provided. Jails reported that this appointment could take place weeks after booking, as it depended on mental health and prescribing provider availability. In addition, while almost a third (*n* = 10) of jails allowed individuals to bring in medications from home, others (*n* = 5) noted explicitly refusing them.

Finally, in most jails (*n* = 18) detention officers, rather than healthcare professionals, reportedly conducted medication administration, i.e., “pill pass,” or accompanied nurses who did. See [citation blinded for submission] for more detailed information about medication administration in jails.

### Challenges Caring for Individuals with Mental Illnesses

Jails reported challenges with high volumes of individuals with mental illnesses, inadequate space and conditions, and managing difficult behaviors.

Jails reported that their limited onsite staffing capacity was not equipped to sustain high volumes of individuals cycling in and out of jail, as one participant described: “The problem we have is there’s so many people come through the door here, like hundreds a day. The nurse can’t possibly see hundreds of people a day…It’s just volume, really, is the problem.” (5, large-sized jail). Specifically, more than half of jails (*n* = 18) reported high volumes of individuals with mental illnesses, regardless of jail size or rurality. As one participant commented: “Most people that come in here, 90 percent of ‘em are mental health.” (26, small-sized jail). At many jails (*n* = 14), participants reported commonly encountering depression, anxiety, PTSD, trauma, bipolar, schizophrenia, or schizoaffective disorders. Other jails described a need for more mental health care to assist individuals with mental illnesses and prevent recidivism, as one participant described:

I really think more mental health would definitely benefit these patients…’cause we have so many that come in on mental health… I feel like if somebody could take the initiative to start helping them, get them on a road to where they could get medications and regularly talk to somebody…about it, maybe, just maybe they wouldn’t come back to jail. (33, small-sized jail)

Jails also reported a limited capacity to provide mental health care. In two jails, participants specifically attributed this to their jails’ inadequate space and housing. As one noted:

…he has [severe] autism… They have him here in medical with us… He’s not going anywhere [because] he’s in [State Division of Family and Children Services] custody, and [they] don’t have nowhere to put him, so they leave him in jail… We’ve got nowhere to put him. (31, medium-sized jail)

Other participants alluded to the negative impact of their jails’ housing conditions on mental health. As one participant described: “You’re gonna put him in a six-by-six cell where he gets no sunlight, no interaction, no nothing, and expect him to get sane?…You’re gonna exacerbate that issue.” (31, medium-sized jail).

Participants in almost a quarter of jails (*n* = 8) described challenges managing the behaviors of individuals with mental illnesses. As one noted: “People are comin’ in, and they’re in psychosis…because they’re not being treated…The acute ones [are] quite hard to manage.” (5, large-sized jail). Reportedly, some of the most challenging behaviors to manage included self-mutilation; refusing to take medications despite severe symptoms; ingesting feces; swallowing inedible objects; inserting objects into body orifices; yelling and spitting; flooding cells; and violence against officers or other incarcerated individuals. One participant noted:

With the woman that we’re trying to [provide mental health treatment for] right now, she is completely in the nude and screams…all the time…No matter what clothing you give [her], if you give her something to lay on, give her [a] blanket, anything you give her goes in her toilet…She has flooded the entire booking department twice now. (27, medium-sized jail)

The same participant continued to describe the steps involved in trying to help her:

She’s been extracted from her cell. Nothing has helped her so far…[The] next step [is for someone from the county]…to come in and observe her and say, “Yeah, she definitely needs to go to a psychiatric ward.” (27, medium-sized jail)

### Discharge Planning Services

Jails reported that discharge planning services in jails were limited in scope, with most services described as providing access to medications and a list of community resources. Central findings and associated challenges are presented below.

### Medications

Participants from most jails (*n* = 21) reported that their discharge process included provision of current medications upon release. Typically a 7-day supply was provided, with reports ranging from a 2- to 30-day supply. One participant described a medication release practice anticipating poor adherence to medical care for mental health medications outside of jail:

When we know that an [individual] is being released, then we will give them…say three to five days’ worth of medication. It depends on what it is. A lot of people are on mental health medication that I know they’re not gonna go to the doctor. (14, Medium-size jail)

Conversely, some jails reported practices that could make it difficult for an individual to continue medications after release. Some jails (*n* = 3) reported that, after release, individuals had to pick up their medication supplies at a community pharmacy, leaving access to medications up to the individual.

### Connecting to Community Services

The most commonly reported method of connecting individuals to community services at release was for the jail to provide a list of community resources. Half of jails (*n* = 17) specifically reported encouraging individuals to continue mental healthcare outside of jail by letting them know where to go. One participant described a typical scenario: “We give them a discharge sheet that gives them the number for mental health and the number for their local health department…It’s like, ‘Here’s the resources. I can’t make you do it, but I highly recommend that you do.’” (14, Medium-sized jail)

While some jails (*n* = 5) reportedly informed individuals about upcoming appointments or made referrals to community mental health agencies, other jails reported that they were limited in the mental health assistance they were able to provide during the discharge planning process. Almost a quarter of participants (*n* = 8) indicated challenges due to a shortage of staff to facilitate the transition as well as not knowing when individuals were getting released. One participant described that, even with some assistance programs, challenges associated with releasing individuals with mental illnesses still remained:

[Local agency] just came to us with a great program. They just got a grant to do all this assistance with providing mental health inmates housing…Well, they come out, they interview these people, and tell them, “Okay. When you get released, you come to [local agency] and we’ll start the process.” Really? Where do they live in the meantime? Where do they go on day one of release? They’re homeless…They don’t have a car. How are they supposed to get to [local agency]? It all sounds great on paper, but reality is just not there. (16, medium-sized jail)

### Lack of continuity of care and subsequent recidivism

When reporting on discharge planning, participants also described serving as safety nets that cared for individuals with mental illnesses with few other options. One third of jails (*n* = 11) explicitly reported on challenges related to poor continuity of mental health care after release. Some participants believed that closed-down mental health hospitals, lack of mental health funding in communities, and low inventory of mental health agencies led to high recidivism in the jailed population with mental illnesses. One participant described this concept and its burden on counties:

There is no other place…The department that’s supposed to take care of them…don’t have anywhere to put them…They closed down all the places that used to house [the] mentally ill because they said that they were inhumane ‘cause they kept them locked up. What are you doing to them now?…The mentally ill have been put out on the streets. Nobody wants to take care of them, so they’re dumping them into jails where there’s no services. There’s no money. There’s nothing, and the counties are being mandated to take care of them. (31, medium-sized jail)

Another participant further explained the lack of transitional community and healthcare resources available post-release and how this related to recidivism:

Most of the health care that they’re getting [is] from a jail setting. So once [incarcerated individual]…comes in with an issue, we’ve medicated him, he’s not been using…We’ve got him healthy. Then he goes back out into the community, he doesn’t have any resources, he doesn’t have any way of getting any resources. He came into the jail; he was mentally ill. Now he’s back out. He’s not taking any medicine. Doesn’t know who to go to…what to do; doesn’t have any housing. And he lands back in the jail. So it’s just the recidivism of inmates cycling in and out…of the jail. And it’s just not enough community resources that are available. (1, large-sized jail)

Other jails (*n* = 7) specifically reported how disruption in post-release medication continuity was associated with recidivism. One participant described: “Sad thing is we’re seeing a lot more…in jail because of mental health…It’s not because they’re under the influence. It’s because they’re off their meds. Get them back on their meds [so] they can be productive in society.” (14, medium-sized jail)

## Discussion

Mental illness is an epidemic across US jails with rates among incarcerated individuals far surpassing those in the general population (4, 5), especially in the Southeastern US (21). This article reports on mental health care challenges and provision from entry to release across multiple jails in several states. In doing so, we address an important gap in the literature by generating new insights about the day-to-day delivery of jail mental health care and by contextualizing prior jail mental health research, which oftentimes has focused on single dimensions of care.

The current study adds to previous evidence of jails reporting mental health care as challenging to manage (18), also finding that jails noted high volumes of individuals with mental health needs and difficulties managing individuals with severe symptoms. Yet many jails reported not having enough resources, specifically onsite mental health staffing, to manage them. In turn, jails reported mental health services as mainly consisting of screening, suicide prevention, and medication management. Initial mental health screenings were often restricted to the detection of suicidality and history of mental health treatment and medications as opposed to current mental health symptoms, and, as other research has found, the use of standardized mental health screening forms was uncommon (16). Additionally, detention officers were often the first point of contact for screenings, sick calls, and suicide prevention monitoring activities. These findings corroborate an earlier study which identified delays in care between the initial health screening and being evaluated by a mental health professional (17); the current study found this occurring more frequently in smaller jails. Delays in receiving mental health medications and medication restrictions and changes were also commonly reported.

Additionally, participants reported poor continuity of mental healthcare and that insufficient community mental health resources greatly contributed to the high rates of re-incarceration among those with mental illnesses. Yet among jails with any discharge planning services, these services were typically limited to providing a partial supply of mental health medications and a list of resources.

### Implications

Although few jails have abundant resources for people struggling with mental illnesses, jails have a constitutional duty to provide adequate healthcare to persons in custody (26). Jails also have an opportunity to care for individuals with mental illnesses in particular, as the majority may have never received mental health treatment (27). However, our findings suggest that current mental health care practices in study jails were mostly insufficient.

### Adoption of Health Standards

First, wide variations in care across jails likely reflects jails’ localized control. Although states and, in some instances, their respective counties have jail health standards or guidance, these often lack detail. Similarly, standards created by national organizations such as NCCHC and the ACA are often broad, and health care accreditation from these organizations is voluntary. As a result, only about 20% of jails are accredited (Amy Panagopoulos, “personal communication,” 5/27/21). Considering that jails can vary greatly by the size of their custodial populations and their associated health care needs, development of “one-size-fits-all” health standards could be challenging—and likely is responsible for the broad language used to craft existing standards. An alternative approach would be to develop multiple sets of standards based on indicators of jail size and needed resources. Regardless, our findings suggest the need for enforceable standards that articulate minimum services such as timely access to providers, medication management and, as addressed below, discharge services.

Specific to mental healthcare, we recommend that the NCCHC standard that jails have 14 days to conduct mental health screenings (28) should be reconsidered as that timeframe may be too wide to address acute issues. In 2019, for example, almost half of those who died by suicide in local jails had been held seven or fewer days at the time of their death (29).

### Opportunity to Identify and Treat Mental Illnesses

Second, findings point to missed opportunities to adequately identify and treat individuals with mental illnesses in jails. The use of mostly unvalidated tools and a reliance on flags related to past treatment or medications could fail to accurately detect such individuals who were merely unable to access mental health care in the community due to poverty and its associated barriers. In fact, one report demonstrated that only 23% of jailed individuals with a mental health problem had received treatment the year before arrest (27). Additionally, with detention officers conducting most initial health screenings, individuals may feel stigmatized (30) or uncomfortable sharing mental health symptoms or histories with officers. This may lead to low detection rates of mental illnesses when trauma and shock are at elevated points and suicide risk is at its highest (6, 31). Gaps to seeing a mental health professional and delays and substitutions in medication provision may also lead to unnecessary suffering among individuals needing timelier and consistent mental health care. This is especially important across the US South and in rural areas with smaller jails, where access to mental health care is especially limited (1, 32).

Improved identification of mental illnesses and suicidality using validated screeners and quicker, less variable follow-up mental health evaluations are needed to adequately identify and more rapidly treat individuals with mental illnesses (33–37). Jails can begin to attain this by focusing on system improvement, i.e., identifying capacity-related issues or areas where healthcare protocols could be improved. One potentially cost-effective solution for mental health evaluations and treatment is the increased use of telemedicine, especially in rural and smaller jails with limited resources (38). While increased funding for mental health services and staffing across jails, especially in smaller jails, is needed to fulfill this, perhaps more essential is leveraging diversion and community programs that prevent incarceration of this population.

### Linkage to Community Mental Healthcare

Lastly, findings suggest that the lack of sufficient mental health discharge planning practices across jails creates missed opportunities for linking individuals with mental illnesses to community resources that have the potential to prevent re-incarceration. Individuals in the criminal legal system are disproportionately made up of people of color and people from low socioeconomic backgrounds who are more likely to be struggling with other disparities such as homelessness, lack of transportation, and loss of employment that could increase recidivism (39–42). Incarceration is also itself a trauma that has negative effects on morbidity and mortality (43–45). Given such disadvantages for this population, challenges jails face caring for this population, plus the exacerbated mental health needs highlighted by COVID-19 pandemic, investment of possible solutions is urgent (46–52). There are initiatives that exist to reduce incarcerations among individuals with mental illnesses, such as the Stepping Up Initiative—a program that helps counties through training, resources, and support to reduce the number of individuals with mental illnesses in jails (53)—however, counties need enhanced assistance and resources for community-based mental health supports and interventions for individuals diverted from jails, such as mental health courts and forensic assertive community treatment teams. Such supports have been shown to be effective at connecting individuals to treatment and reducing recidivism (54–59). One strategy currently underway in North Carolina, which other Southeastern states could consider adopting, is the use of Medicaid expansion dollars to enhance mental health services for incarcerated individuals and in communities; this has the potential to improve mental health care treatment and prevent recidivism for this population (32, 60).

### Limitations

There were several limitations to our study. First, although results are commonly presented alongside numbers, such results should be interpreted with caution. Numerical comparisons across interviews cannot be made; instead, readers should interpret common themes across jails. We were not able to uniformly ask every question on our interview guide or obtain screening forms from every jail, which could limit generalizability of findings. Also, since we interviewed jails in the Southeastern US, we cannot generalize our findings to other regions and jails. Though the sample was not all randomly selected, we aimed to include jails that represented a diverse range of experiences. In addition, our findings do not include recent challenges addressing mental illnesses in the wake of increased mental health needs and exacerbated challenges to meet such needs within the jail environment affected by COVID-19. Importantly, we did not ask for patient perspectives, which could have uncovered vastly dissimilar and crucial information.

## Conclusion

Jails have become the *de facto* caretakers of some of our most marginalized and vulnerable groups—individuals with mental illnesses—who are disproportionately represented in carceral settings. Yet jails are inappropriate facilities to care for such individuals. Findings from interviews across the Southeastern US highlight the widespread challenges jails face having to provide mental healthcare with inadequate resources, and jail practices that create missed opportunities to identify, treat, and facilitate linkage to community care at release. Findings also point to the difficulties endured by individuals with mental illnesses incarcerated in jails and the need to reconsider current incarceration practices for this population. Findings also enhance existing information on mental health screening and services across multiple jails and states, which provides valuable context that can be used to inform improvements in mental health care policy across the nation. Jails, communities, and policymakers have a responsibility to enhance supports that reduce recidivism and increase access to community mental healthcare for individuals living with mental illnesses.

## Figures and Tables

**Table 1:** Demographic characteristics.

Participants (*N*= 38)	*n*	%
Gender		
Female	25	66
Male	3	8
Missing^a^	10	26
Race		
White	21	55
Black/African American	7	18
Missing	10	26
Age		
<35	5	13
35-55	16	42
>55	6	16
Missing	11	29
Role		
Regional healthcare manager	4	11
Local nurse/healthcare administrator	30	79
Jail administrator	11	4
Educational Degree		
RN	39	15
LPN	37	14
Other	11	4
Missing	13	5
Years in current position		
< 1	13	5
1–5	45	17
6–10	16	6
10+	18	7
Missing	8	3

a Data not collected at time of interview.

**Table 2:** Jail characteristics.

	*n*	%
Jail size (capacity) ^a^		
Large (999+)	6	18
Medium (100–999)	21	62
Small (0–99)	7	21
Accreditation		
Yes	8	25
No, follows guidelines	10	24
No, or unsure	8	27
Missing	8	24
Over Capacity^b^	6	18
Jail County Rurality Level^c^		
Urban	17	50
Rural	15	44
Completely rural	3	9

a Jail size as determined by the Bureau of Justice Statistics (BJS) and based on the 2006 and 2013 BJS Census of Jails.

b Overcapacity defined as daily population (at time of interview) that exceeds jail capacity.

c This table reflects data on the percentage of the county population living in rural areas as of the 2010 Census. Counties with less than 50 percent of the population living in rural areas are classified as mostly urban; 50 to 99.9 percent are classified as mostly rural; 100 percent rural are classified as completely rural.

**Table 3:** Mental Health Staffing and Supplementary Services.

Mental Health Staffing	Availability	Coverage	Distinction by Jail Size
Overall Mental Health Staffing	Among all study jails (*n* = 34), participants, nearly half (*n* = 15) reported the absence of routine onsite mental health staffing of any kind (e.g., counselors, psychiatric prescribing providers)	Most jails (*n* = 21) reported that onsite mental health staffing of any kind was limited to once a week or less, only in crises, or nonexistent Of jails with any onsite mental health staff available (*n* = 19), most had (*n* = 13) onsite staffing at least 2 times per week; six jails had onsite staffing once per week	The majority of jails (*n* = 9) with full-time or nearly full-time onsite mental health counseling tended to be larger jails with a capacity of more than 500 incarcerated individuals The majority of jails (*n* = 17) with limited (once a week or less) or no onsite mental health counseling tended to be jails with capacities of 200 or fewer incarcerated individuals
Medication Management Staffing	Less than a third of jails (*n* = 10) reported regularly-scheduled onsite prescribing mental health providers (e.g. psychiatric nurse practitioners or psychiatrists) available for medication management and referrals Other jails (*n* = 9) reported using a psychologist or mental health counselor to evaluate individuals and make medication recommendations to a prescribing provider not specifically trained in prescribing psychiatric medications (e.g. physician, physician’s assistant, or nurse practitioner)	Most jails (*n* = 25) reported limited onsite prescribing mental health provider coverage at once a week or less, or none at all Almost all jails (*n* = 9) reporting onsite prescribing mental health provider coverage reported it as occurring more than once a week	
Onsite Counseling Staffing	Most jails (*n* = 18) reported regularly- scheduled onsite counseling staff (e.g., psychologist, social worker, or therapist) Almost half of jails (*n* = 15) reported no onsite mental health counseling coverage or only occurring on an as- needed basis (e.g., for court evaluations) or in crisis circumstances (e.g., suicide assessments) Other jails (*n* = 11) reported full-time or nearly full-time onsite mental health counseling	Most jails (*n* = 21) reported limited onsite counseling staff coverage at once a week or less, or none at all Most jails with regularly-scheduled onsite counseling staff (*n* = 11) reported onsite counselor coverage at more than once a week
Telemedicine- Based Mental Health Staffing	Some jails (*n* = 9) utilized telemedicine for medication management and/or mental health counseling in addition to or instead of onsite staff	Most jails providing telemedicine services (*n* = 6) reported it as occurring once a week or less Most jails (*n* = 24) reported that a prescribing provider or a mental health counselor was available 24/7 via phone or telemedicine to discuss patients deemed to be in crisis
Community and Prison System Resources	Other jails (*n* = 7) relied on offsite community mental health agencies to supplement a lack of onsite mental health staff One state utilized a program in which jails unable to provide sufficient health care onsite could, for a fee, transfer a limited number of individuals to the state prison system for care; half of jails (*n* = 6) using this program reported using it specifically for individuals with severe mental illnesses	Community mental health service coverage was often described as ad hoc and was used to address episodes considered to be psychiatric emergencies

## Data Availability

The datasets used and/or analyzed during the current study are available from the corresponding author on reasonable request.
